# Effect of Potassium Chlorate on the Treatment of Domestic Sewage by Achieving Shortcut Nitrification in a Constructed Rapid Infiltration System

**DOI:** 10.3390/ijerph15040670

**Published:** 2018-04-04

**Authors:** Qinglin Fang, Wenlai Xu, Zhijiao Yan, Lei Qian

**Affiliations:** 1 State Key Laboratory of Geohazard Prevention and Geoenvironment Protection, Chengdu University of Technology, Chengdu 610059, China; txgsfy@163.com; 2 State Environmental Protection Key Laboratory of Synergetic Control and Joint Remediation for Soil and Water Pollution, Chengdu University of Technology, Chengdu 610059, China; yanzhijiaocdut@126.com (Z.Y.); qianleicdut@yeah.net (L.Q.)

**Keywords:** shortcut nitrification, constructed rapid infiltration system, potassium chlorate inhibition, domestic sewage

## Abstract

A constructed rapid infiltration (CRI) system is a new type of sewage biofilm treatment technology, but due to its anaerobic zone it lacks the carbon sources and the conditions for nitrate retention, and its nitrogen removal performance is very poor. However, a shortcut nitrification–denitrification process presents distinctive advantages, as it saves oxygen, requires less organic matter, and requires less time for denitrification compared to conventional nitrogen removal methods. Thus, if the shortcut nitrification–denitrification process could be applied to the CRI system properly, a simpler, more economic, and efficient nitrogen removal method will be obtained. However, as its reaction process shows that the first and the most important step of achieving shortcut nitrification–denitrification is to achieve shortcut nitrification, in this study we explored the feasibility to achieve shortcut nitrification, which produces nitrite as the dominant nitrogen species in effluent, by the addition of potassium chlorate (KClO_3_) to the influent. In an experimental CRI test system, the effects on nitrogen removal, nitrate inhibition, and nitrite accumulation were studied, and the advantages of achieving a shortcut nitrification–denitrification process were also analysed. The results showed that shortcut nitrification was successfully achieved and maintained in a CRI system by adding 5 mM KClO_3_ to the influent at a constant pH of 8.4. Under these conditions, the nitrite accumulation percentage was increased, while a lower concentration of 3 mM KClO_3_ had no obvious effect. The addition of 5mM KClO_3_ in influent presumably inhibited the activity of ammonia-oxidizing bacteria (AOB) and nitrite-oxidizing bacteria (NOB), but inhibition of nitrite-oxidizing bacteria (NOB) was so strong that it resulted in a maximum nitrite accumulation percentage of up to over 80%. As a result, nitrite became the dominant nitrogen product in the effluent. Moreover, if the shortcut denitrification process will be achieved in the subsequent research, it could save 60.27 mg CH_3_OH per litre of sewage in the CRI system compared with the full denitrification process.

## 1. Introduction

**Figure 1:**
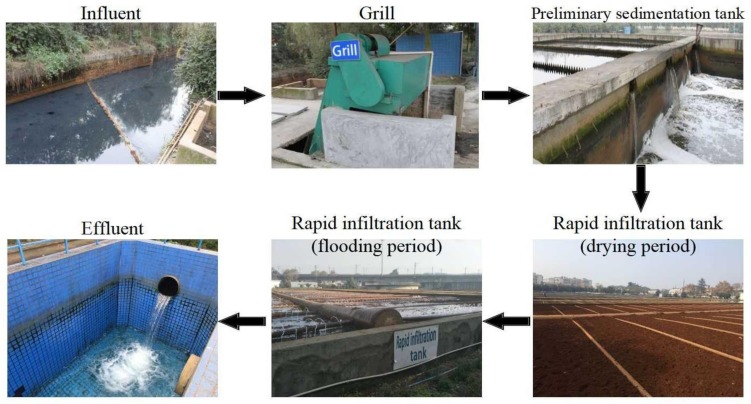
Practical engineering of the Phoniex River constructed rapid infiltration (CRI) system operated successfully for 12 years in Chengdu, China.

Sewage treatment technology for domestic sewage and polluted surface water treatment in small towns—a constructed rapid infiltration (CRI) system—is a new sewage biofilm treatment technology put forward by Zhong Zuoshen et al. [1]. It presents both advantages of a sewage rapid infiltration land treatment system and a constructed wetland system [2]. A CRI system is mainly composed of a feeding tank, grill, preliminary sedimentation tank, rapid infiltration tank, and outlet system. A CRI system adopts the dry-wet (alternate running of feeding and drying in the CRI system) alternating operation mode and uses natural river sand, coal gangue, natural gravel, etc., to replace natural soil as the filling medium to improve the hydraulic load to 1.0–1.5 m/day [3]. Pictures of a practical example of a CRI system are shown in Figure 1. The removal mechanism of the CRI system is to use the filling medium and microorganisms grown on the filling medium to adsorb, intercept, and decompose the pollutants in sewage [4]. Especially, since the CRI system has the unique structure and feeding mode, its filling medium has the aerobic, facultative, and anaerobic environment to grow abundant microorganism to allow for efficient sewage treatment [5]. As the previous practice showed, a CRI system has a significant effect on the treatment of domestic sewage in small towns [6], whose removal rates of CODcr (chemical oxygen demand determined by potassium dichromate method), NH_4_^+^-N, suspended solid (SS), and linear alkylbenzene sulfonates (LAS) could reach above 85%, 90%, 95%, and 95%, respectively, and has the advantages of being less energy-intensive, more environmentally-friendly, and has a remarkable economic benefit compared with the conventional treatment systems [7]. Although a CRI system has a good removal effect of NH_4_^+^-N, due to its anaerobic zone it lacks the carbon sources for denitrification and the condition for nitrate retention [8], the concentration of nitrate in effluent is so high that the total nitrogen (TN) removal rate can only reach upwards of 10–30% [9].

To enhance the nitrogen removal performance of the CRI system, the methods of adding external carbon sources, optimizing the packing structure [10], and changing the water feeding patterns [1] were adopted. However, those methods were all based on the full nitrification–denitrification process, making it difficult to overcome the problem of carbon source consumption and reduction of denitrifying bacteria activity during long-term operation, and were also difficult to popularize in the actual engineering due to their complex operating process.

**Figure 2:**
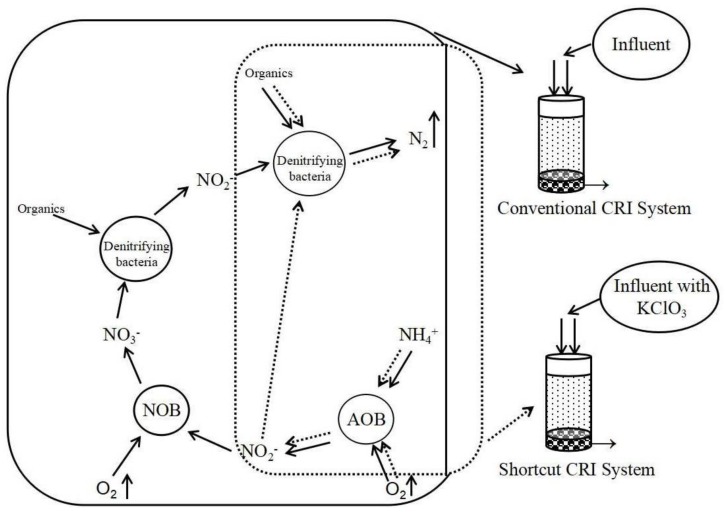
Comparison of the full nitrification–denitrification process and shortcut nitrification–denitrification process (→ represents the process of full nitrification–denitrification; ⇢ represents the process of shortcut nitrification–denitrification).

Shortcut nitrification–denitrification is a novel biofilm nitrogen removal process which allows oxidation of ammonia to nitrite, but no further oxidation to nitrate and reduces nitrite into nitrogen gas directly to achieve nitrogen removal in the system. As Figure 2 shows, compared to the full nitrification–denitrification process, the shortcut nitrification–denitrification process reduces two reaction steps, which are “NO_2_^−^ → NO_3_^−^”and “NO_3_^−^ → NO_2_^−^”. Thus, it will present the advantages of saving oxygen and requiring less organic matters. However, it can also be seen from Figure 2, for shortcut nitrification–denitrification to be employed, the key point is to achieve shortcut nitrification. In other words, the system must accumulate and maintain enough nitrite, which is produced by ammonium-oxidizing bacteria (AOB) and, at the same time, inhibit or wash out nitrite-oxidizing bacteria (NOB), which would oxidize the produced nitrite to nitrate [11]. The conditions required to inhibit nitrite oxidization can be established with high concentrations of ammonium, a low concentration of dissolved oxygen, a high concentration of free nitrous acid, a relatively high temperature (30–35 °C) and a high pH (8–9). So far, shortcut nitrification has been achieved in various systems, such as aerated constructed wetlands [12], a sequencing batch reactor (SBR) [13] and submerged biofilters [14], all of which resulted in high nitrite accumulation percentages. The use of specific inhibitors can further improve shortcut nitrification. For example, Xu et al. [13] studied the effect of hydroxylamine addition on shortcut nitrification in SBR, and Chen et al. [15] used this same inhibitor in a CRI; both found nitrite accumulation percentages reaching more than 90%. Sukru and Erdal [14] and Cui et al. [16] found that increasing salinity could further promote the accumulation of nitrite. Moreover, Ge et al. [17] showed that low concentrations (4 mg/L) of chlorine could improve the nitrite accumulation percentage to reach 60–70%. Already in 1957 chlorate was described as a specific inhibitor of NOB: chlorate could inhibit the growth of autotrophic nitrite oxidizers at low concentration (4.2 × 10^−3^ M) and completely inhibit nitrite oxidation at high concentrations (1.7 × 10^−2^ M) [18]. Subsequent studies reported that the addition of chlorate could result in nitrite to become the dominant product of NO_x_ in the effluent, by allowing AOB activity while inhibiting NOB. For instance, Xu et al. [11] showed that the addition of chlorate to aerobic granules resulted in a 90% increase of nitrite accumulation in the effluent. Other studies showed that chlorate inhibited the oxidation of nitrite to nitrate, but it did not affect the oxidation of NH_4_^+^ to NO_2_^−^ [19]; likewise, Xu et al. [11] found that oxidation of NH_4_^+^ to NO_2_^−^ was not severely inhibited by chlorate. Such studies showed that shortcut nitrification can be achieved effectively by the addition of specific inhibitors, including chlorate, but the effect of adding potassium chlorate (KClO_3_) in CRI system has not yet been studied in detail. 

In this study, we tested whether potassium chlorate could improve the performance of shortcut nitrification and removal efficiency of pollutants in a CRI system under experimental conditions and prospected the benefits of achieving shortcut denitrification.

## 2. Materials and Methods

### 2.1. Experimental Design

**Figure 3:**
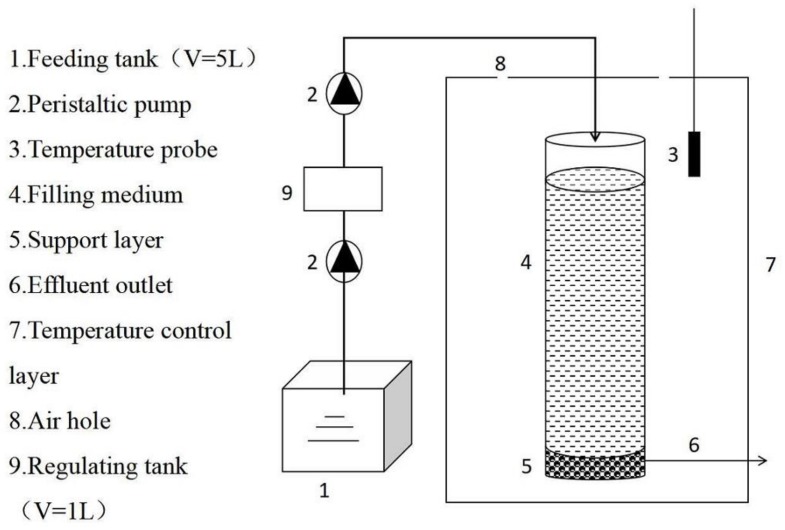
Experimental CRI system. CRI: constructed rapid infiltration.

Four separate CRI columns were constructed using PVC (polyvinyl chloride) (diameter 8 cm, height 30 cm) in the laboratory under controlled conditions. The temperature was kept constant at 34.2 ± 0.64 °C by constructing a temperature-controlling box around the CRI columns (Figure 3). The filling medium of the columns consisted of two functional layers: a 5 cm deep supporting layer consisting of pebbles (5.0–10.0 mm) and gravel (3.0–4.0 mm) at the bottom, a 20 cm deep treatment layer filled with 90% river sand (0.25–0.30 mm), 5% marble sand (1.0–2.0 mm), and 5% zeolite sand (1.5–1.7 mm) on the top of the supporting layer. The influent sewage was lifted by a peristaltic pump so that it entered at the top of the column, moved through the packing medium vertically, and left by the outlet where water quality was measured. 

### 2.2. Sewage and Operational Conditions

The influent sewage used in this study was a mixture of synthetic sewage and domestic sewage, the synthetic sewage was mainly made up of glucose, CH_3_COONa, (NH_4_)_2_SO_4_, KH_2_PO_4_, Na_2_CO_3_, and peptone and was refilled every three days. The water quality parameters are shown in Table 1. The whole experiment lasted for 110 days. The sewage was fed into the system by a dry-wet alternating operation mode as follows: water feeding was allowed twice daily with a hydraulic load of 0.6 m/day, each feeding time would last for 1.5 h, each drying time would last for 10.5 h, and the water flow was 200 mL/h. The system was operated for 70 days until the removal percentages of ammonium nitrogen (NH_4_^+^-N) in effluent of all columns reached to 88%, which indicated the biofilms had formed successfully in the CRI system. 

**Figure 4:**
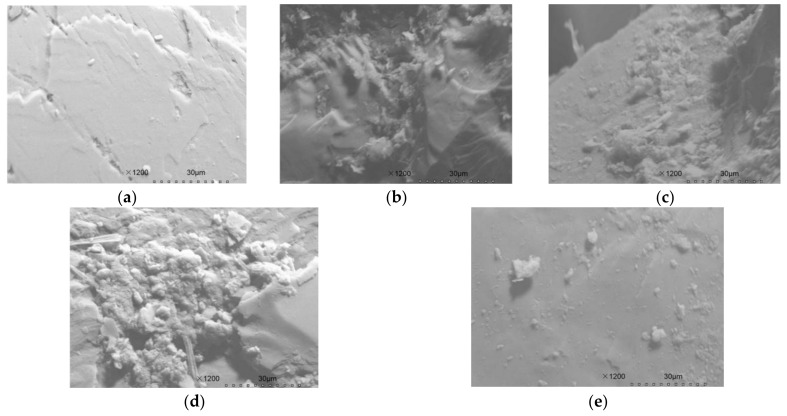
Scanning electron microscope (SEM) images of filling medium (sand) in the CRI columns after 70 days of operation. (**a**) blank filling medium; (**b**–**e**) filling medium (formed with biofilm) of Tests 1–4.

In order to investigate the effect of potassium chlorate inhibition and pH control, the experimental columns were used as individual Tests. Test 1 was the control treatment not receiving additions, the pH of influent of Tests 2–4 was adjusted to 8.4 by addition of NaOH solution. Moreover, in Test 3, KClO_3_ was added to the influent at a final concentration of 5 mM while, in Test 4, a concentration of 3 mM KClO_3_ was used. Both the NaOH solution and KClO_3_ were added and mixed in the regulating tank after it loaded with 600 mL sewage from feeding tank. Moreover, the scanning electron microscope (SEM) pictures (Figure 4) of the filling medium (sand) were taken on day 70, which could further show the situation of biofilm formation on the filling medium of tests 1–4. As we can see from Figure 4, the blank filling medium (picture a) which was not fed sewage, can hardly investigate the microbial flora attachment. However, the filling medium of Tests 1–4 (pictures b–e) which were fed sewage for 70 days had an obvious microbiological attachment, which indicated that biofilms were formed successfully in the filling medium of Tests 1–4.

### 2.3. Analytical Methods

Water samples from influent and effluent were collected every two days, filling medium samples were collected after biofilm formed successfully (on day 70). Concentration of COD in the sewage was determined using the potassium dichromate method (in a strong acid solution, a certain amount of potassium dichromate is used to oxidize the reducing substances in the water sample, then, ferroin (indicator) is added to the excess potassium dichromate before it is titrated with ammonium ferrous sulfate solution, and the oxygen consumption of the reductive substance in the water sample is calculated according to the amount of ammonium ferrous sulphate); the concentration of nitrogen in the form of ammonium was determined by Nessler’s reagent colorimetric method (an alkaline solution made of mercuric iodide and potassium iodide reacted with ammonium nitrogen would generate reddish brown complex, the absorbance of the complex which was measured at 420 nm (visible light) and is proportional to the content of ammonium nitrogen), nitrate (NO_3_^−^-N) by UV spectrometry (the concentration of nitrate nitrogen can be quantified by the absorption value of nitrate ion at a wavelength of 220 nm, however the dissolved organic matter was absorbed at both 220 nm and 275 nm, while the nitrate ion was not absorbed at 275 nm, therefore another measurement is made at 275 nm to correct the absorption of nitrate nitrogen), nitrite (NO_2_^−^-N) by molecular absorption spectrophotometry (in phosphoric acid medium, nitrite is reacted with para-aminobenzene sulfonamide to produce diazonium salt, then, coupling with *N*-(1-naphthyl) ethylenediamine to produce red dye, finally, determining the absorbance of production at 540 nm (visible light)), and total nitrogen (TN) by UV spectrometry (at 120–124 °C basic potassium persulfate solution is used to convert nitrogen-containing compounds into nitrate in the water sample, then, the ultraviolet spectrophotometry method is used to determine the absorbency of the sample at 220 nm and 275 nm respectively, the corrected absorbance (A) is calculated according to the formula (A = A_220_ − 2A_275_) and is proportional to the total nitrogen content), using standard procedures [20]. The nitrite accumulation percentage was calculated as the ratio of NO_2_^−^/(NO_2_^−^ + NO_3_^−^) × 100% [12]. 

### 2.4. Scanning Electron Microscope Detection

The biofilm of the filling medium was prepared by the glutaraldehyde fixation method [21] and observed by using a scanning electron microscope (SEM) (S-3000N, Hitachi Limited, Tokyo, Japan). The filling medium samples were fixed with 2.5% glutaraldehyde for 15 h and then rinsed in distilled water three times. Subsequently, the samples were dehydrated with series of ethanol (30%, 50%, 70%, 85%, 95%) for one time, and 100% ethanol for two times (20 min/time). After rinsing twice (20 min/time) with isoamyl acetate, the prepared samples were natural dried for 12 h. Finally, the dewatered samples were sputter-coated with gold and observed with SEM. 

## 3. Results and Discussion

### 3.1. Effect of Potassium Chlorate on Removal Efficiency of Ammonium Nitrogen

The removal efficiency of nitrogen in the form of ammonium in the CRI system was compared between the controls (with and without pH adjustment) and after the addition of two concentrations of KClO_3_ to the influent. Removal efficiency was calculated as the difference in concentration between influent and effluent (influent concentration minus effluent concentration) divided by the concentration in influent.

**Figure 5:**
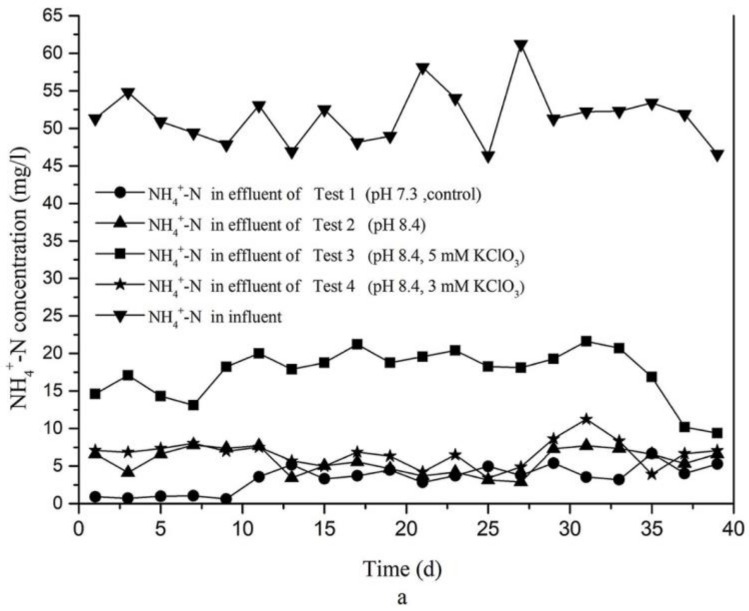

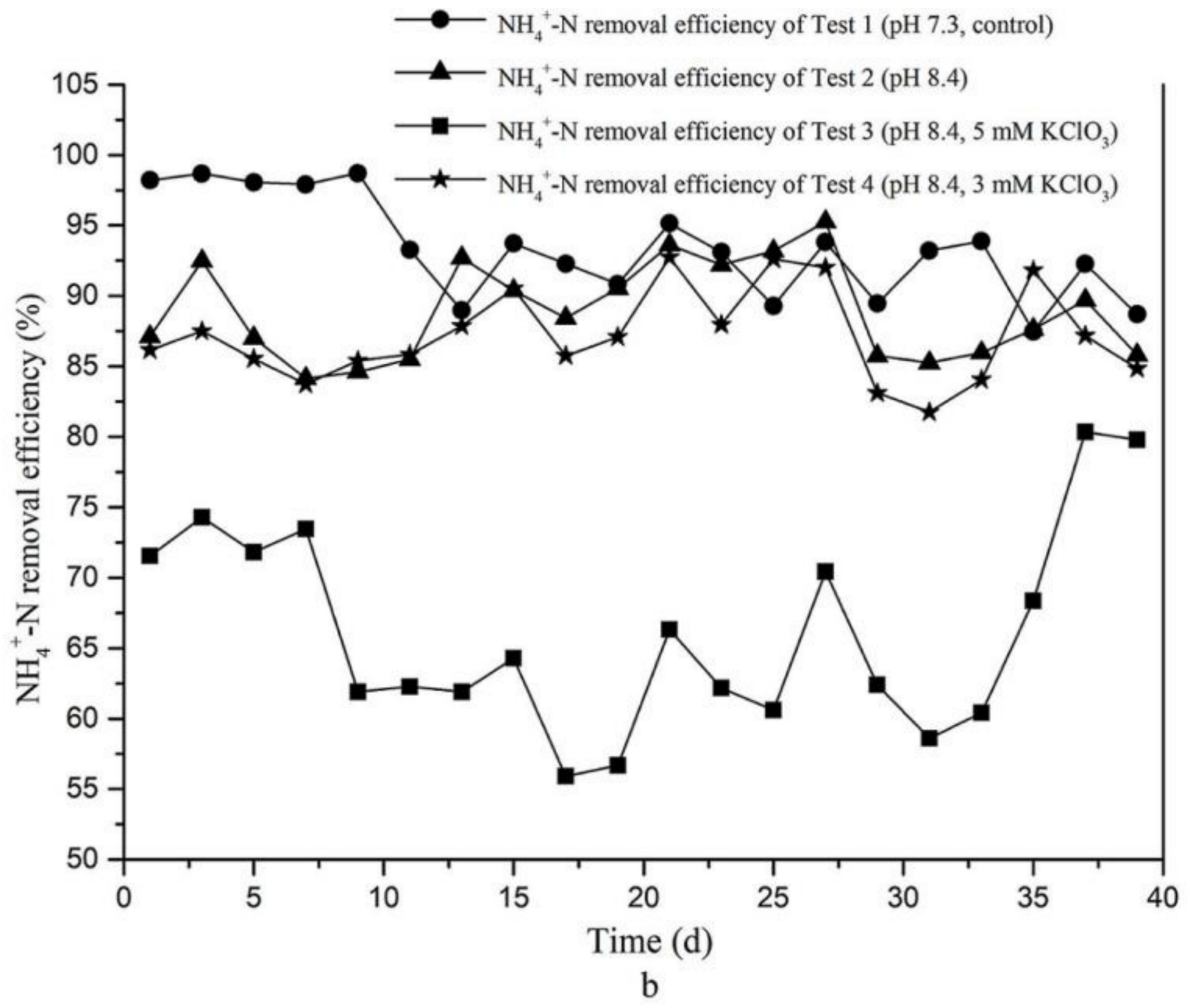
Ammonium-nitrogen removal. (**a**) Absolute concentrations of NH_4_^+^-N in effluent and influent; (**b**) Ammonium-nitrogen removal efficiency (in %) of the four experimental tests of CRI.

Adjustment of the influent pH to 8.4 of Test 2 only had a minor effect on ammonium nitrogen removal during the first 10 days (Figure 5), the reason may be that the AOB need time to adapt the new pH environment in the system. There was no difference in removal efficiency between Test 4 (pH 8.4, 3 mM KClO_3_) and Test 2 (pH 8.4), as both reached approximately 87% removal on average (Figure 5). However, in presence of 5 mM KClO_3_ (Test 3), the NH_4_^+^-N removal efficiency was reduced, though it still reached 66% on average. Xu et al. [11] also found that oxidation NH_4_^+^ to NO_2_^−^ was slightly inhibited by chlorate. This is most likely the chlorate has a slight inhibition of AOB activity, as a result of which NH_4_^+^-N oxidation efficiency was less efficient. 

### 3.2. Effect of Potassium Chlorate on Nitrate Accumulation in a CRI System

**Figure 6:**
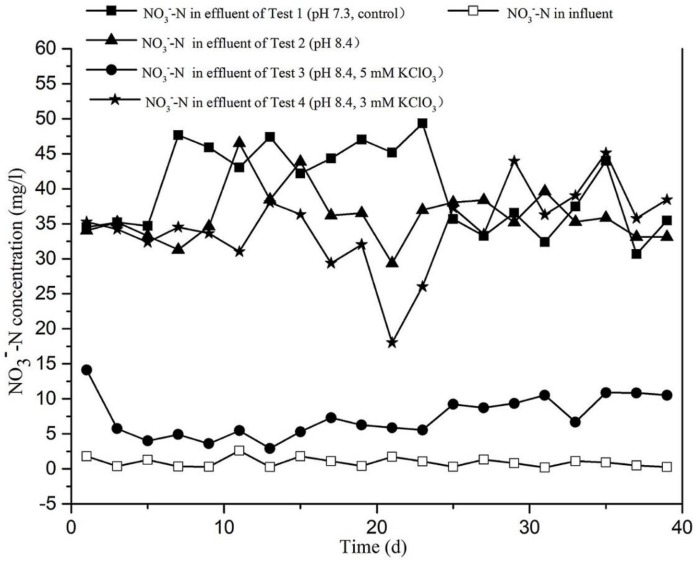
The nitrate-nitrogen concentration in influent and effluent in the four experimental tests of CRI system.

As can be seen in Figure 6, there was no significant difference between Test 2 (pH 8.4), resulting in a nitrate concentration of on average 36.24 mg/L, and Test 4 (pH 8.4, 3 mM KClO_3_), resulting in 34.51 mg/L. Very similar results were obtained for the control in which the pH of the influent had not been adjusted (Test 1, pH 7.3). In contrast, Test 3 (pH 8.4, 5 mM KClO_3_) resulted in much lower nitrate concentrations of approximately 7.39 mg/L on average, which represented an 80% reduction compared to the control. As shown, the nitrate concentration in effluent of Test 3 was reduced within 48 h after addition of 5 mM KClO_3_ and reached a minimum of 2.92 mg/L on day 13. This result shows that addition of 5 mM KClO_3_ to the influent was able to strongly prevent the oxidation of nitrite, a condition that favours the accumulation of nitrite and is desired for shortcut nitrification achievement.

### 3.3. Effect of Potassium Chlorate and pH on Nitrite Accumulation in a CRI System

**Figure 7:**
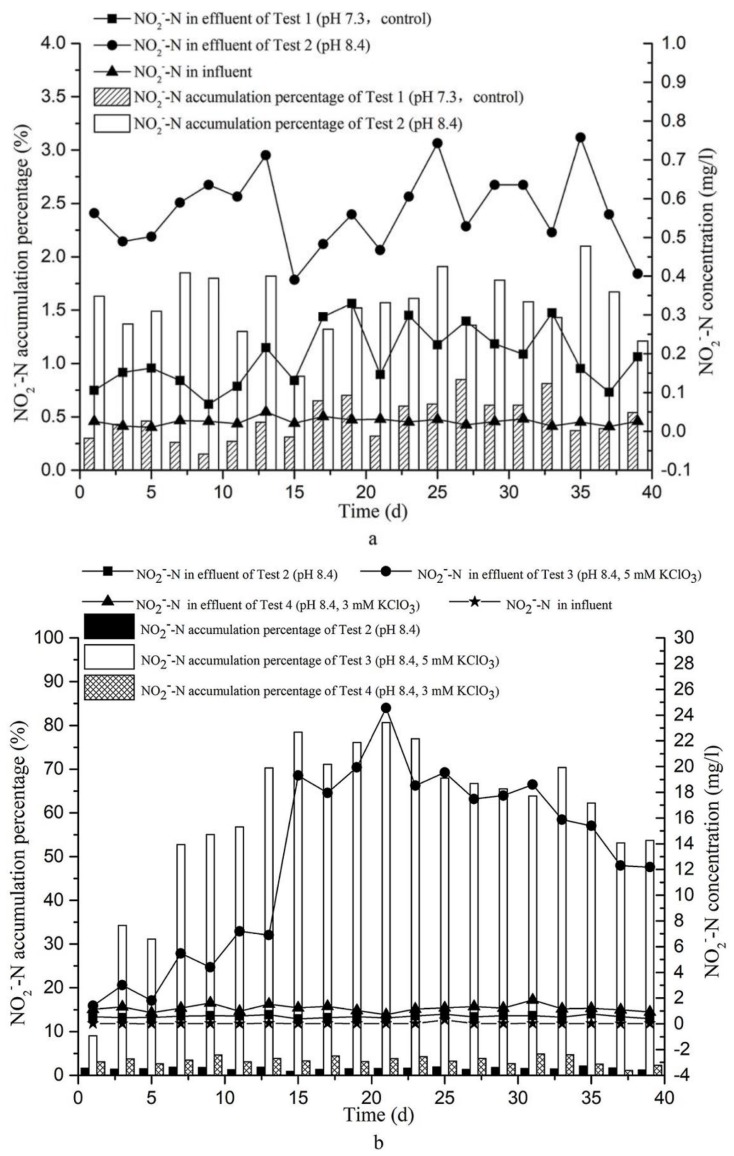
Nitrite accumulation in effluent. (**a**) Effect of pH on nitrite accumulation percentage (bars) and concentration (curves) in the effluent of Test 1 (pH, 7.3) and Test 2 (pH 8.4); (**b**) The nitrite accumulation percentage (bars) and concentration (curves) in effluent of Tests 2–4.

Previous studies have described that the pH of the influent is a decisive factor for inhibiting NOB activity. For instance, Banashri [19] described that nitrite accumulation can be improved at high pH (8–9). Glass and Silverstein [22] observed a significant increase of nitrite accumulation (250, 500 mg/L) in sequencing batch reactors when wastewater pH was increased during nitrification (pH 7.5, 8.5, respectively). Thus, we adjusted the influent sewage pH to 8.4 of Test 2 and observed (Figure 7a) that the average nitrite accumulation percentage of Test 2 (pH 8.4) was 1.5%, which was slightly higher than that of Test 1 (0.50%, pH 7.3). Nevertheless, this increase was too weak to support shortcut nitrification. Thus, a pH of 8.4 is, by itself, insufficient to enable effective shortcut nitrification in a CRI system.

The average nitrite accumulation percentage in our tests are shown as bars in Figure 7b. As can be seen, these percentages were very low in Test 2 (1.56% on average) and Test 4 (3.43% on average), but much increased in Test 3, resulting in 59.80% accumulation percentages on average. Thus, the addition of 5 mM KClO_3_ strongly supported accumulation of nitrite in the test CRI system. Combined with the data presented in Figure 5, Figure 6 and Figure 7, it can be concluded that, whereas nitrate was the dominant product in effluent of Tests 1, 2, and 4, nitrite was the dominant nitrogen product of Test 3, as a result of effective nitrite oxidation inhibition.

As apparent in Figure 7b, the nitrite accumulation percentage in effluent of Test 3 increased sharply during the first seven days (from, initially, 9.02% to 52.76%) and further increased to reach a plateau of up to 80% during days 15–23. The nitrite concentration peaked at day 21 at 24.54 mg/L. After this, the nitrite accumulation percentage slightly decreased, but still reached 53% at day 39. The reason may be that the long-term addition of the chlorate will cause a slight inhibition of AOB activity. However, the result also indicates that shortcut nitrification can be not only be achieved, but also maintained in the tested CRI system by the addition of 5 mM KClO_3_ in the influent at a pH of 8.4.

### 3.4. Prospects for the Achievement of Shortcut Nitrification–Denitrification in a CRI System

Xu et al. [11] mentioned that the shortcut nitrification–denitrification process could save 40% of carbon source consumption, compared with the full nitrification–denitrification process. Chen [23] observed that an increased COD concentration (51.3, 69.3, 73.3 mg/L) in the effluent in biological filters when the nitrite percentage (NO_2_^−^/(NO_2_^−^ + NO_3_^−^) × 100%) in the influent was increased (0%, 50%, 80%, respectively). As we can see from Figure 7, the shortcut nitrification could be achieved successfully in the CRI system by adding 5 mM KClO_3_ in Test 3, and its mean concentration and accumulation percentage of nitrite could reach to 12.98 mg/L and 59.80%, respectively. Thus, the accumulation of nitrite in Test 3 could provide electron acceptor for the subsequent shortcut denitrification. However, Chen et al. [15] found that the removal efficiency of COD could reach 90% during shortcut nitrification in the CRI system. Wang et al. [8] mentioned that carbon source, nitrate/nitrite and anaerobic environment are essential for denitrification in the CRI system since most of the denitrifying bacteria are facultative anaerobic and use organic matters as carbon sources under the anoxic condition to provide energy. However, in this study, we calculated that the removal percentage of CODcr of four tests all reached more than 91%, and the mean concentration of residual CODcr of Test 3 was only around 7.28 mg/L, which was too low to support the subsequent shortcut denitrification. Therefore, if a shortcut denitrification experiment will be conducted, an external carbon source is required to be added to the influent. Methanol, ethanol, acetic acid, and cellulose were all studied as external carbon sources for denitrification in previous studies. Yan et al. [24] found that methanol is easily biodegraded and used by denitrifying bacteria and the denitrification rate of methanol is very high. Zhang [25] found that the complete removal of nitrogen in effluent can be achieved by adding sufficient methanol in denitrification process. Gómez et al. [26] found that methanol is an ideal carbon source for denitrification. Thus, if methanol (CH_3_OH) is chosen as an external carbon source for subsequent shortcut denitrification, the equations of full denitrification (Equation (1)) and shortcut denitrification (Equation (2)) are shown as follows:

NO_3_^−^ + 1.08CH_3_OH + 0.24H_2_CO_3_ → 0.056C_3_H_7_O_2_N + 0.47N_2_ + 1.68H_2_O + HCO_3_^−^ (1)

NO_2_^−^ + 0.67CH_3_OH + 0.53H_2_CO_3_ → 0.004C_3_H_7_O_2_N + 0.48N_2_ + 1.23H_2_O + HCO_3_^−^ (2)

According to Equations (1) and (2) and the data from Figure 6 and Figure 7, the mean nitrate concentration of Test 2 (pH 8.4) and Test 3 (pH 8.4, 5 mM KClO_3_) are about 36.24 mg/L and 7.39 mg/L, respectively, the mean nitrite concentration of Test 2 and Test 3 are about 0.57 mg/L and 12.98 mg/L, respectively. If the subsequent shortcut denitrification will be achieved, the dosage of CH_3_OH used for Test 2 denitrification will consume 98.38 mg CH_3_OH per litre of sewage during the operating period, but, Test 3 only needs 38.11 mg CH_3_OH per litre of sewage for denitrification and shortcut denitrification, which was only 38.73% of the consumption of Test 2. Moreover, an anaerobic environment is also important for the denitrification in the CRI system. Fan et al. [1] added a sub-section intake and overflow pool in the CRI system simulated columns and found this method will increase the total nitrogen removal efficiency to 64.8%. Therefore, if the shortcut nitrification–denitrification process in the CRI system is implemented in the subsequent research, not only will the external carbon source be added in the denitrification section, but a saturated water layer will also be constructed in the bottom of the denitrification section to improve the total nitrogen removal performance of the CRI system. Furthermore, although, the chlorate is easily biodegraded by nitrate reductase in an organic-rich environment, the appropriate amounts of reductant also need to be added into the reactor to fully eliminate potential pollution when the shortcut nitrification process ended [11]. Thus, although achievement of shortcut nitrification–denitrification process in the CRI system will present many advantages, such as improving the denitrification rate, simplifying the reaction process, and saving carbon source consumption, there is still much research work needed to be done towards applying this new technology in a practical project. 

## 4. Conclusions

(1) The addition of 3 mM KClO_3_ to influent at a constant pH of 8.4 is not sufficient to inhibit that of NOB so that shortcut nitrification does not take place in the CRI system.
(2) Adjusting the pH of influent to 8.4 alone did not contribute much to establish shortcut nitrification in CRI.
(3) Although, the addition of 5 mM KClO_3_ in influent could both inhibit the activity of ammonia-oxidizing bacteria (AOB) and nitrite-oxidizing bacteria (NOB), the inhibition of NOB was so strong that made the NO_2_^−^-N to be the dominant product of total oxidized nitrogen in effluent for a long period, showing that shortcut nitrification could be achieved and maintained successfully in a CRI system.
(4) According to the data of nitrate and nitrite in Figure 6 and Figure 7, the consumption of external carbon source (CH_3_OH) for subsequent denitrification was calculated and analysed by using Equations (1) and (2), the results showed that the consumption of carbon source (CH_3_OH) of Test 3 (pH 8.4, 5 mM KClO_3_) was only 38.73% of the consumption of Test 2 (pH 8.4). Therefore, compared with conventional sewage treatment methods, achievement of the shortcut nitrification–denitrification process in the CRI system will take both the advantages of the CRI system and shortcut nitrification–denitrification process; it will not only have a unique structure and feeding mode to construct aerobic, facultative, and anaerobic environments for microorganism enriching in the filling medium, but also improve the denitrification rate and save the carbon source consumption during the reaction process.

## Figures and Tables

**Table 1:** Water quality parameters of influent.

Water Quality Parameters	Mean Concentration (mg/L)
Chemical Oxygen Demand (COD)	245.22 ± 27.11
NH_4_^+^-N	53.93 ± 3.81
NO_3_^−^-N	1.15 ± 0.67
NO_2_^−^-N	0.14 ± 0.09
Total Nitrogen (TN)	55.35 ± 6.01
pH	7.3 ± 0.14 (control), 8.4 (Tests 2–4)
Temperature (°C)	34.2 ± 0.64
